# Secreted factors from olfactory mucosa cells expanded as free-floating spheres increase neurogenesis in olfactory bulb neurosphere cultures

**DOI:** 10.1186/1471-2202-9-24

**Published:** 2008-02-18

**Authors:** Perrine Barraud, Xiaoling He, Maeve A Caldwell, Robin JM Franklin

**Affiliations:** 1 Department of Veterinary Medicine, University of Cambridge, Madingley Road, Cambridge CB3 0ES, UK; 2 Cambridge Centre for Brain Repair, University of Cambridge, Madingley Road, Cambridge CB3 0ES, UK; 3 Henry Wellcome Laboratories for Integrative Neuroscience and Endocrinology, Dorothy Hodgkin Building, Bristol University, Whitson Street, Bristol BS1 3NY, UK

## Abstract

**Background:**

The olfactory epithelium is a neurogenic tissue comprising a population of olfactory receptor neurons that are renewed throughout adulthood by a population of stem and progenitor cells. Because of their relative accessibility compared to intra-cranially located neural stem/progenitor cells, olfactory epithelium stem and progenitor cells make attractive candidates for autologous cell-based therapy. However, olfactory stem and progenitor cells expand very slowly when grown as free-floating spheres (olfactory-spheres) under growth factor stimulation in a neurosphere assay.

**Results:**

In order to address whether olfactory mucosa cells extrinsically regulate proliferation and/or differentiation of immature neural cells, we cultured neural progenitor cells derived from mouse neonatal olfactory bulb or subventricular zone (SVZ) in the presence of medium conditioned by olfactory mucosa-derived spheres (olfactory-spheres). Our data demonstrated that olfactory mucosa cells produced soluble factors that affect bulbar neural progenitor cell differentiation but not their proliferation when compared to control media. In addition, olfactory mucosa derived soluble factors increased neurogenesis, especially favouring the generation of non-GABAergic neurons. Olfactory mucosa conditioned medium also contained several factors with neurotrophic/neuroprotective properties. Olfactory-sphere conditioned medium did not affect proliferation or differentiation of SVZ-derived neural progenitors.

**Conclusion:**

These data suggest that the olfactory mucosa does not contain factors that are inhibitory to neural stem/progenitor cell proliferation but does contain factors that steer differentiation toward neuronal phenotypes. Moreover, they suggest that the poor expansion of olfactory-spheres may be in part due to intrinsic properties of the olfactory epithelial stem/progenitor cell population.

## Background

The olfactory epithelium is a neurogenic tissue containing a population of olfactory receptor neurons (ORNs) that are renewed throughout adulthood [1]. Precursors of the ORNs reside in the olfactory epithelium as a population of transit amplifying cells called globose basal cells (GBCs) [2,3]. Adjacent to the GBCs and in contact with the basement membrane of the olfactory epithelium, are the horizontal basal cells (HBCs). In normal conditions, HBCs are relatively quiescent, but following injury and ORN degeneration they proliferate and can give rise to GBCs and non-neuronal lineage cells, and thereby regenerate the olfactory epithelium [4,5]. In contrast to the subventricular zone (SVZ), hippocampus and the olfactory bulb (all of which are located within the cranium), the olfactory epithelium represents an accessible source of stem/progenitor cells for autologous transplantation for central nervous system (CNS) repair that can be isolated by simple biopsy without profoundly altering the sense of smell [6,7]. However, few methods to expand olfactory stem and progenitor cells under serum-free conditions have been described and as a result, the therapeutic values of olfactory stem and progenitor cells remains uncertain.

Attempts to expand olfactory stem and progenitor cells under growth factor stimulation in serum-free condition according to the well-established free-floating cell aggregate CNS neurosphere culture method [8] have been described in two studies [9,10]. Although olfactory stem cells from the developing mouse generate primary spheres, they fail to generate secondary spheres after cell dissociation or passage [10]. Whether this is due to intrinsic factors, inappropriate extrinsic factors or a combination of both is not known. We have reasoned that if extrinsic factors were involved then they should be present in olfactory-sphere conditioned medium and that these factors would affect the proliferative properties of a neural stem/progenitor cell known to readily generate secondary spheres. In order to address this, we cultured neonatal mouse neural progenitor cells isolated from the olfactory bulb and the SVZ as neurospheres [11] in a medium supplemented with either olfactory mucosa cell-conditioned medium or with fresh bFGF/EGF-containing medium. Our data demonstrate that olfactory mucosa cells produce soluble factors that affect olfactory bulb neural progenitor cell differentiation toward a neuronal cell fate without affecting their proliferation but have no effect on SVZ neurosphere cell proliferation and differentiation. We found that soluble factors from olfactory mucosa cells increased neurogenesis, but not gliogenesis (although were able to decrease expression of the astrocyte marker glial fibrillary acidic protein (GFAP)) from olfactory bulb-derived spheres. These data suggest that the olfactory mucosa does not contain factors that are inhibitory to neural stem/progenitor cell proliferation but does contain factors that steer differentiation toward neuronal phenotypes. Moreover, they suggest that the poor expansion of olfactory-spheres may be in part due to intrinsic properties of the stem/progenitor cell population.

## Results

### Olfactory mucosa conditioned medium does not affect proliferation of olfactory bulb- or subventricular zone-derived neural progenitor cells

**Figure 1:**
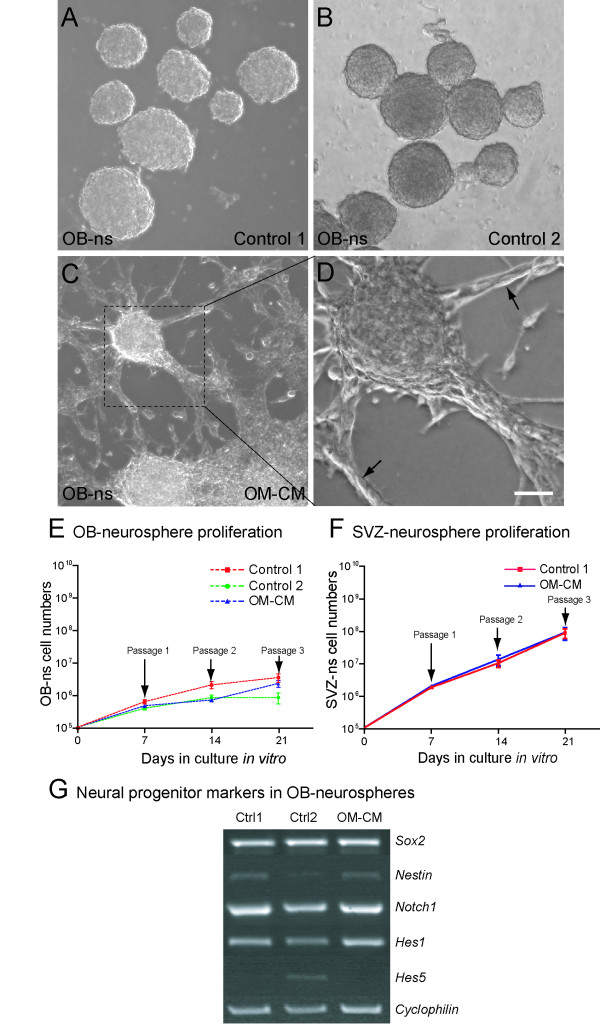
Effects of olfactory mucosa conditioned medium on olfactory bulb (OB-ns) and subventricular zone neurosphere cell (SVZ-ns) proliferation. (A-D) Photomicrographs of 7 day old olfactory bulb neurospheres expanded in bFGF/EGF (control 1 shown in A and control 2 shown in B) and in OM-CM (C and D). (D) High magnification of photomicrograph C revealing cells located away from the core of the neurosphere (arrows). (E-F) Graphs showing proliferation curves of control cultures (control 1 and control 2) and neurosphere cells derived from the olfactory bulb in (E) and from the subventricular zone (F) cultured in OM-CM. (G) Semi-quantitative RT-PCR showing expression level of the neural stem and progenitor cell markers in OB-ns in control cultures and OM-CM. Scale bars: 100 μm in A-C and 50 μm in D.

In an earlier study, we demonstrated that dissociated olfactory mucosa cells from neonatal mice can generate heterogeneous primary spheres (or "olfactory-spheres") under bFGF and EGF stimulation [9]. These cultures comprise two types of olfactory-spheres based on size and cellular composition; small diameter sphere (≈ 25–75 μm) containing sustentacular cells and HBCs, and large diameter spheres (100–400 μm) containing HBCs, GBCs, and olfactory ensheathing cells [9]. Since primary olfactory-spheres, despite containing HBCs and GBCs, fail to generate secondary spheres after passage, we asked whether cells within the spheres released soluble factors inhibiting proliferation of CNS progenitor cells. To address this we used neonatal mouse olfactory bulb and SVZ as a source of neural stem and progenitor cells and expanded them as neurospheres (OB-ns and SVZ-ns, respectively) in the following culture conditions. The first culture condition was prepared using 50% olfactory-sphere conditioned medium (OM-CM) and 50% "expansion medium" containing fresh bFGF and EGF (making a final concentration of bFGF and EGF of 10 ng/ml). Since OM-CM also contains bFGF and EGF (the same expansion medium was used to expand the olfactory-spheres; see material and methods section), we generated two control cultures consisting of "expansion medium" supplemented with fresh bFGF and EGF at two final concentrations: 20 ng/ml (control 1) or 10 ng/ml (control 2). OB-ns were expanded for 7 div in OM-CM or control conditions 1 and 2. Properties of OB-ns at 7 div prior to cell dissociation are shown in Figure 1. While most OB-ns were free-floating in control cultures (control 1 see Fig 1A, and control 2, see Fig 1B), OB-ns expanded in OM-CM attached to the flask and many cells were identified away from the spheres, suggestive of their having migrated (Fig 1C,D).

In order to examine the effects of OM-CM on neural progenitor cell proliferation we counted total cell numbers using the trypan blue exclusion method after the first, second and third passage and compared cell numbers from the OB-ns and the SVZ-ns expanded in the presence of OM-CM with control cultures (Fig 1E,F). We found no significant differences in the cell numbers between OM-CM and control cultures at passages 1, 2 or 3, indicating OM-CM did have an additive affect on neural progenitor cell proliferation.

To address whether soluble factors present in the OM-CM affected expression neural stem/progenitor cell markers Sox2, nestin, Notch1 and the Notch regulators Hes1 and Hes5, we looked at RNA expression of these markers in OB-ns cells expanded in OM-CM and control cultures using semi-quantitative RT-PCR [12-15]. We found minor differences in the expression level of *nestin*, *Notch1*, *Hes1 *and *Hes5 *between the two control cultures: *Hes5 *transcripts were only detected in control 2 but not in control 1 and expression levels of *nestin*, *Notch1 *and *Hes1 *was lower in control 2 compared to control 1 (Fig 1G). However, similar expression levels of all these neural stem/progenitor cell genes were detected between control 1 and OM-CM cultures indicating that olfactory mucosa soluble factors did not contain factors affecting expression levels of these markers (Fig 1G).

These data indicate that factors present in OM-CM do not influence proliferation of neural progenitor cells nor do they affect expression level of gene encoding neural stem/progenitor cell markers. OM-CM therefore does not contain inhibitors of progenitor proliferation which might account for the slow expansion of growth properties of olfactory-spheres.

### Olfactory mucosa cells produce soluble factors that affect neurogenesis from olfactory bulb-derived progenitors but not from subventricular zone-derived progenitors

**Figure 2:**
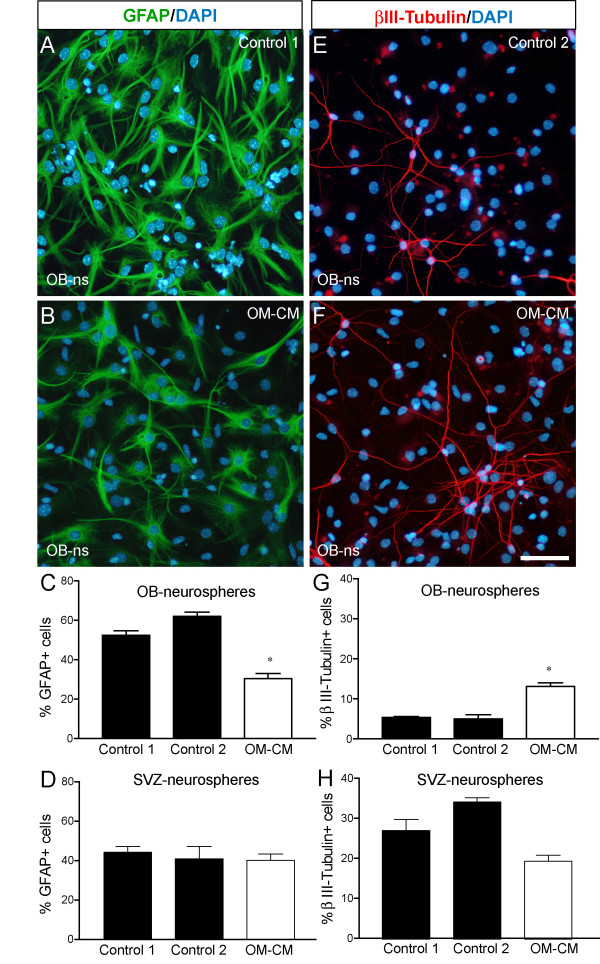
Glial fibrillary astrocytic protein (GFAP) and βIII-tubulin expression in differentiated olfactory bulb-derived (OB-ns) and subventricular zone-derived (SVZ-ns) neurospheres. (A-B) Photomicrographs showing GFAP-expression in control OB-ns (A) and OM-CM cultures (B). Proportions of GFAP-expressing cells in OB-ns and SVZ-ns cultures are shown in histogram graphs in C and D, respectively. (E-F) Photomicrographs showing βIII-tubulin expression in control (E) and OM-CM cultures (F). Proportions of βIII-tubulin-expressing cells in OB-ns and SVZ-ns cultures are shown in histogram graphs in G and H, respectively. Scale bar in A, B, E, F: 50 μm.

To examine whether soluble factors present in OM-CM could affect OB-ns and SVZ-ns differentiation into neurons and astrocytes we subjected neurospheres expanded in OM-CM or control conditions to differentiation conditions for 7 div. No significant differences in cell densities were observed in control OB-ns cultures *versus *OM-CM cultures after 7 days differentiation (72.3 ± 17.03 cells/mm^2 ^in control 1 vs. 62.53 ± 12.74 cells/mm^2 ^in OM-CM cultures; P value>0.05). Neurons were identified using anti-βIII-tubulin antibodies and astrocytes were identified using anti-GFAP antibodies. Our data revealed that OB-ns expanded with OM-CM contained significantly fewer GFAP-positive cells than control cultures (36.5 ± 2.5% in OM-CM *vs*. 62 ± 2.2% in control 2, P < 0.001; see Fig 2A,B,C) whereas OM-CM did not affect the proportion of GFAP-expressing cells in SVZ-neurosphere cultures (Fig 2D). In contrast, total cell numbers of βIII-tubulin^+ ^cells significantly increased when the OB-ns were expanded in OM-CM compared to control cultures (13.1 ± 0.9% in OM-CM *vs*. 5 ± 1% in control 2, P < 0.001; see Fig 2E,F,G) whereas in SVZ-ns, OM-CM did not significantly decrease the proportion of βIII-tubulin-expressing cells when compared to control 1 cultures (Fig 2H). Total numbers of olfactory ensheathing cells (identified with anti-low affinity NGF receptor antibody) or oligodendrocyte-lineage cells (identified with anti-galactocerebroside antibody) were not significantly different between control and OM-CM OB-ns cultures, both representing approximately 1 to 2% of the total cells (data not shown). In differentiated OB-ns cultures, the nuclei of astrocytes (GFAP^+ ^cells) were large and round compared to the small ovoid nuclei of neurons (βIII-tubulin^+ ^cells) (Fig 2A,B and 2E,F, respectively). A large proportion of cells with large and round nuclei (characteristic of astrocytes) were GFAP^- ^indicating that the decrease in GFAP-expressing cells observed in OM-CM cultures was likely to be due to a decrease in GFAP expression rather than a decrease in total numbers of astrocytes. Therefore, these data indicate that expanded olfactory mucosa cells produce soluble factors that increase the numbers of neurons 2.5 fold but decrease GFAP expression in OB-ns-derived astrocytes.

**Figure 3:**
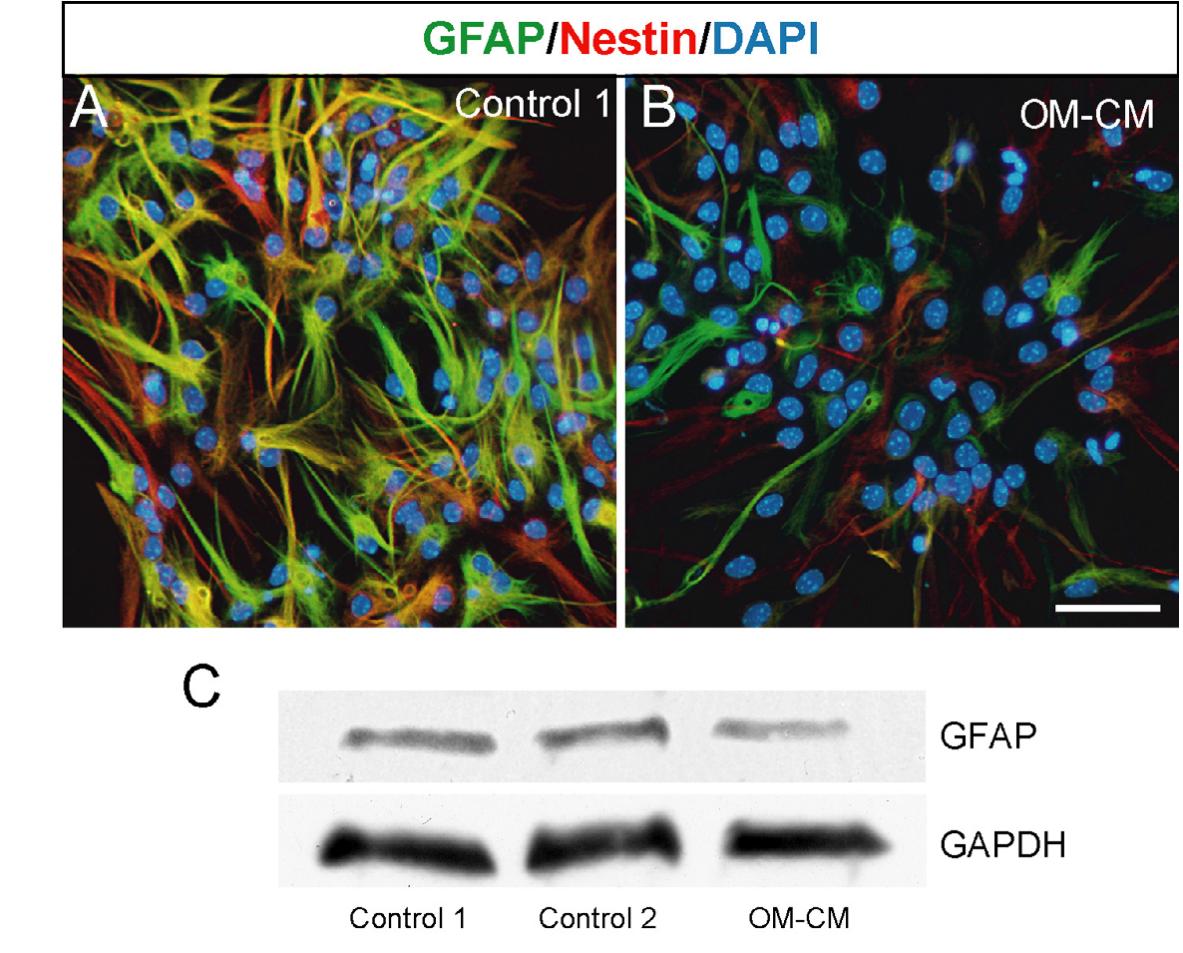
Glial fibrillary astrocytic protein (GFAP) and nestin expression in differentiated olfactory bulb neurospheres expanded in control medium (A) and in OM-CM (B). Western blot showing expression level of GFAP and GAPDH in control cultures (control 1 and 2) and OM-CM cultures in C. Scale bar in A, B: 50 μm.

We next focused our study on olfactory bulb neural progenitor cells and addressed whether this decrease in GFAP expression observed in differentiated OB-ns could be related to a delay in astrocyte differentiation by immunolabelling differentiated preparations with anti-GFAP combined with anti-nestin, a marker for neural stem/progenitor cells [12]. In both control and OM-CM cultures, nestin-expressing cells had an astrocytic morphology (large and round nuclei) similar to GFAP-expressing cells indicating that decreased number in GFAP-expressing cells was likely to be due to a decrease in expression of GFAP rather than a delay in astrocytic differentiation (Fig 3A,B). To confirm this, we quantified expression level of GFAP in both controls and OM-CM cultures using western blot (Fig 3C). The expression level of GFAP was lower in OM-CM cultures compared to control 1 and 2 (Fig 3C). These data indicate that soluble factors present in OM-CM increase neurogenesis, but do not affect gliogenesis. However, some factors in OM-CM decrease the number of strongly GFAP-expressing astrocytes.

### OM-CM decrease the number of GABAergic but not calretinin-containing neurons from the olfactory bulb

**Figure 4:**
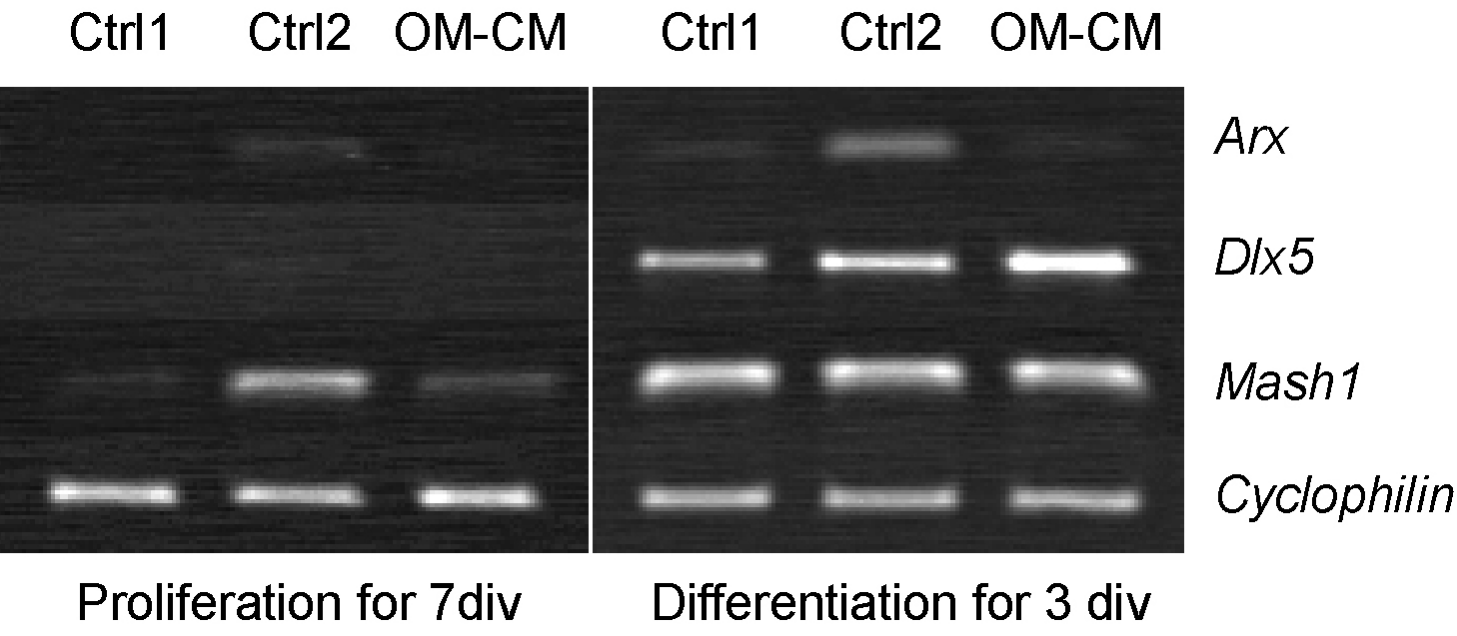
Semi-quantitative RT-PCR comparing expression level of genes encoding olfactory bulb interneuron markers between controls and OM-CM cultures in proliferation and differentiation conditions.

Neuronal progenitors present in the olfactory bulb originate from the SVZ and migrate via the rostral migratory stream to the olfactory bulb where they integrate into the local neuronal circuitry (for review see [16]). Several transcription factors have been shown to participate in olfactory bulb interneuron specification, migration, and differentiation. Mash1 is highly expressed in the subpallial ventricular zone (and SVZ) and give rise to Dlx5^+ ^cells, a marker for differentiating calretinin-containing neurons and a subset of GABAergic neurons [17-20]. Arx is involved in the migration and differentiation of GABAergic interneurons [21]. We evaluated expression levels of mRNA for *Mash1*, *Dlx5 *and *Arx *in both proliferating and differentiating OB-ns cultures using semi-quantitative RT-PCR (Fig 4). Under proliferation conditions, transcripts for *Mash1 *were found in all culture conditions but at different levels of expression; the highest level of expression was found in control 2 and the lowest, in control 1 (Fig 4). Expression level of *Mash1 *mRNA was higher in OM-CM than control 1 cultures. In differentiating cultures, transcripts for *Dlx5 *were found at a higher level in OM-CM compared to control cultures; no differences were found in the expression level of *Mash1 *and *Arx *between control 1 and OM-CM cultures (Fig 4). These data therefore suggest that some soluble factors may affect differentiation of calretinin-containing neurons and a subset of GABAergic interneurons.

**Figure 5:**
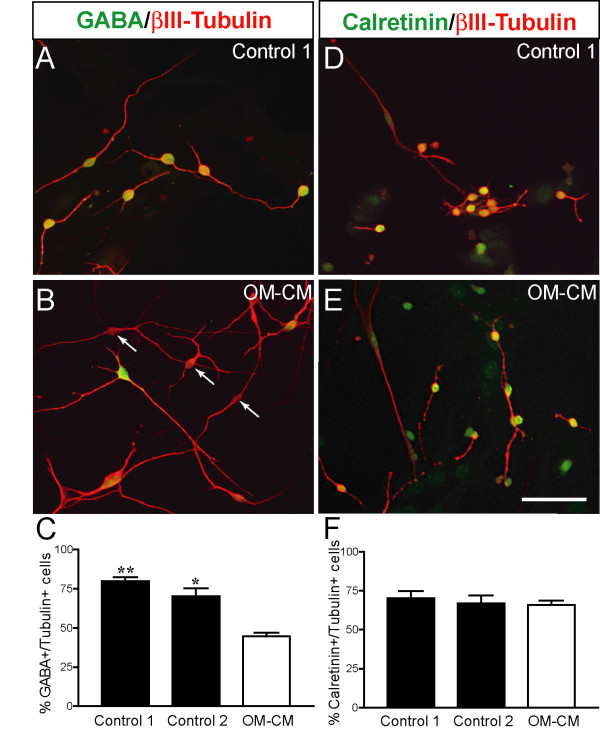
γ-amino butyric acid (GABA), calretinin and βIII-tubulin expression in differentiated olfactory bulb neurosphere cells. (A-B) Photomicrographs showing GABA and βIII-tubulin-expression in control (A) and OM-CM cultures (B) and calretinin and βIII-tubulin-expression in control (D) and OM-CM cultures (E) which proportions are shown in histogram graph in C and F, respectively. Scale bar in A, B, D, E: 50 μm.

Periglomerular cells within the olfactory bulb are identifiable by their expression GABA, tyrosine hydroxylase (TH), calbindin and calretinin; GABAergic, calretinin and calbindin represents the three mains subsets, whereas TH-expressing interneurons represent a small subset within the GABAergic population (for review see [22]). To confirm effects of OM-CM on periglomerular cell subset development OB-ns were differentiated for 7 div and immunolabelled with anti-GABA, anti-TH, anti-calretinin, and anti-calbindin (Fig 5). In our cultures TH^+ ^or calbindin^+ ^cells were not identified (data not shown) whereas a large number of cells were found to be GABA^+ ^or calretinin^+ ^(Fig 5A,B and 5D,E respectively). Cultures were colabelled with βIII-tubulin and either anti-GABA or anti-calretinin (Fig 5A,B, and Fig 5D,E). Cell quantification of βIII-tubulin^+^/GABA^+ ^cells in OM-CM and control cultures indicated that among the GABA-containing cells there were significantly less βIII-tubulin^+^/GABA^+ ^cells in OM-CM compared to control cultures (44.78 ± 2.2% in OM-CM *versus *80 ± 2.4% in control 1; Fig 5C). However, no differences were found in number of calretinin/βIII-tubulin^+ ^cells between OM-CM and control cultures (Fig 5F). Therefore, these data indicate that OM-CM contains soluble factors that favour differentiation of neurons that express calretinin but not GABA.

### Neurotrophic/neuroprotective factors produced by olfactory-spheres

**Figure 6:**
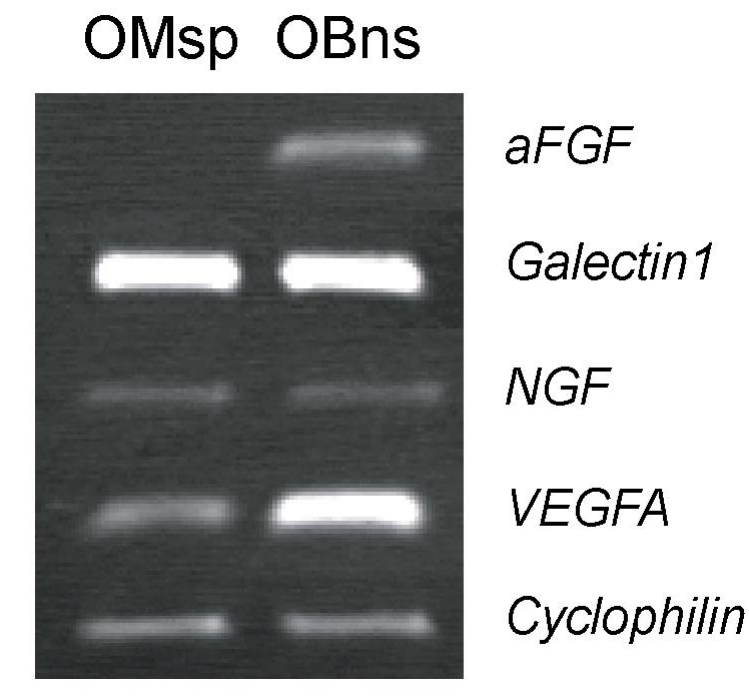
Semi-quantitative RT-PCR comparing expression level of genes encoding neuroprotective factors in olfactory mucosa derived spheres (OM-sp) and in olfactory bulb neurospheres (OB-ns).

CNS stem/progenitor cells can replace lost neurons and glial cells in addition to producing several soluble factors with neurotrophic and/or neuroprotective effects [23-25]. To assess whether olfactory-spheres produce soluble factors with potential to promote neural repair, we compared expression level of mRNA encoding soluble factors between olfactory-spheres and neurospheres, using semi-quantitative RT-PCR (Fig 6). We evaluated mRNA expression levels of selected factors with neuroprotective and neurotrophic effects: acidic FGF, Galectin-1, NGF and VEGF-A (for review see, [26-29]). Our data, shown in Figure 6, revealed that transcripts encoding Galectin-1, NGF, and VEGF-A (except aFGF) were all identified in both olfactory-spheres and neurospheres. However, mRNA encoding aFGF were only detected in the neurospheres and not in the olfactory-spheres. Transcripts encoding for NGF and Galectin-1 were identified at similar level in both the olfactory-spheres and neurospheres. By contrast, transcripts of *vegf-a *gene were found at a higher level in neurospheres compared to olfactory-spheres. Therefore, these data indicate that both olfactory-spheres have the potential to produce some but not all neuroprotective factors released by neurospheres.

## Discussion

In the mature olfactory epithelium the generation of new ORNs is under genetic and autocrine/paracrine controls [30-32]. In this study we investigated whether soluble factors released by olfactory-spheres that are composed of HBCs, GBCs, olfactory ensheathing cells, and sustentacular cells [9] affected neural progenitor cell proliferation and/or differentiation. Using olfactory bulb as a source of neural stem/progenitor cells and a neurosphere assay, we found that factors produced by olfactory-spheres 1) increased adherence of neural stem/progenitor cells without affecting their proliferation and 2) increased neurogenesis while decreasing the proportion of GFAP-expressing cells. When the SVZ was used as source of neural stem and progenitor cells, factors secreted by olfactory mucosa cells had no effects on their proliferation or differentiation.

Transcripts encoding factors known to have mitogenic effects on neural progenitor cells like VEGF and Galectin-1 [33-36] were detected in our olfactory-spheres indicating that neural stem/progenitor mitogens are likely to be present in the OM-CM. The fact that we did not observe any significant differences in total cell numbers (between control and OM-CM cultures) could be explained by the following assumptions: 1) mitogens are present at a too low concentration to readily affect progenitor proliferation, 2) their activity is dependent of the status of the proteins (*e.g*. monomeric or dimeric form, oxidized) and therefore mitogens present in the OM-CM may be inactive, or 3) olfactory bulb and olfactory epithelial neural progenitor cells may require distinct factors cooperating with bFGF and/or EGF to stimulate their proliferation.

During the sphere expansion phase we found that OM-CM stimulated neurospheres to adhere to the culture flask. Several proteins have been reported to promote neural stem/progenitor cell adhesion without altering their proliferation and/or promoting their differentiation when expanding under growth factor stimulation. Such proteins include poly-ornithine, fibronectin and laminin [37-39]. Poly-ornithine and laminin have been used to expand under serum-free conditions neural stem/progenitor cells from fetal and adult mouse brains [37,39]. Furthermore, disruption of β1-integrin expression – a receptor for both laminin and fibronectin – on neural stem/progenitor cells dramatically decreased their adherence in addition to their proliferation when stimulated by both bFGF and EGF [38]. All these data together indicates that extracellular proteins like laminin and/or fibronectin may be produced by olfactory-spheres and such proteins may increase adherence of olfactory bulb-derived neurospheres without altering their proliferation.

In addition to increase cell adhesion during expansion, OM-CM affected neurogenesis when neurospheres were subjected to differentiation. Upon differentiation for 3 div, expression level of the neuronal marker Dlx5 was increased when cultures were exposed to OM-CM during their expansion compared to control cultures. Moreover, when neurospheres were differentiated for 7 div, total number of βIII-tubulin^+ ^cells increased when the cells were exposed to OM-CM during their expansion compared to control cultures. It is probable that during the expansion phase and in the early phase of differentiation (3 days after differentiation was induced), a component contained in OM-CM may have affected survival and/or stimulated proliferation of a small subset of proliferating neuronal precursor cells. Supporting this conjecture is the presence of mRNA encoding Dlx5 (a transcription factor involved in the generation of bulbar calretinin-containing neurons and a subset of GABAergic interneurons; see [17-20]) that was expressed at a higher level in differentiating OM-CM cultures than differentiating control cultures. In our study we found that the proportion of βIII-tubulin^+^/GABA^+ ^cells decreased when neurospheres were primed with OM-CM prior to differentiation compared to unprimed cultures (control cultures) whereas proportion of βIII-tubulin^+^/calretinin^+ ^cells were similar in all three culture conditions. However, since there is a two fold increase in βIII-tubulin^+ ^cells in OM-CM cultures compared to controls these data indicate that soluble factors present in OM-CM do not affect differentiation toward GABAergic neurons but favours the calretinin-containing neurons. Tyrosine hydroxylase (a limiting enzyme in the biosynthesis of dopamine) and calbindin were not detected in our cultures by immunocytochemistry and we cannot conclude whether OM-CM affects the dopaminergic and/or calbindin-containing subclasses of periglomerular cells.

In this study we found that olfactory bulb neurospheres expanded in the presence of OM-CM during their expansion for 7 div 1) attached to the culture flask, 2) gave rise to more neurons than control cultures, 3) contained less GABAergic neurons than control cultures and 4) contained less GFAP-expressing cells than control cultures. These data are similar to the data obtained from Tarasenko and colleagues [40]. These authors generated neurospheres from human fetal forebrain and cultured them in a serum free-medium supplemented with heparin, bFGF and laminin prior to differentiation. Addition of laminin while neurospheres proliferated, increased their adherence to the flask, increased neurogenesis, decreased expression of GFAP and finally decreased total numbers of GABA^+^/βIII-tubulin^+ ^cells [40]. Given similarities between our results and Tarasenko et al.'s data, it is tempting to propose that laminin may be one of the candidate proteins that affect cell adhesion and neurogenesis in both olfactory bulb neurospheres and olfactory mucosa spheres. In our previous study we found that olfactory mucosa cells attached to the bottom of the flask whereas olfactory bulb neurospheres cultured in the same expansion medium did not adhere to the flask. Moreover, olfactory mucosa cells were expanded as free-floating spheres when plated on culture flask coated with a polymer in order to prevent their adhesion ([9]; see material and methods). Recently, it has been reported that anti-GBC-3, an antibody raised to a cell surface antigen specifically expressed by GBCs, recognized a non-integrin precursor of a receptor for laminin [41]. In the olfactory system, laminin in addition to galectin-1 are abundant along the olfactory nerve pathway [42]. Laminin and galectin-1 are both secreted by olfactory ensheathing cells and both create a permissive environment for the olfactory receptor axons to project to specific areas of the bulb [42].

Based on our previous data [9] and considering results from other groups [40-42], we propose the following scenario: olfactory mucosa cells expanded as olfactory-spheres may secrete laminin into the medium. Secreted laminin may increase adherence of the olfactory-spheres to the bottom of the flask and even promote differentiation of the GBCs given that a large number of cells were positive for the neural cell adhesion molecule (NCAM) a marker for differentiating neurons. As a result laminin may decrease proliferation potential of olfactory stem/progenitor cells when expanded as free-floating spheres.

Although olfactory-spheres differed from neurospheres for their limited potential to self-renew (*i.e*. to generate new spheres after subsequent dissociation or passage), they demonstrated potential to produce soluble factors with neuroprotective effects. In this study, mRNA encoding Galectin-1, VEGF and NGF were detected in both olfactory-spheres and neurospheres indicating that olfactory mucosa cells expanded under bFGF/EGF stimulation can secrete factors known to have effects on neural cell survival, axonal growth and even axonal regeneration (for review see [43-45]). We therefore provide in this study the first evidence that olfactory stem/progenitor cells expanded as free-floating spheres can potentially promote axonal regeneration and/or neuroprotection by soluble factors they secreted.

What implications do our data gathered from mouse tissue have for human-derived cells? Recent studies performed in rodent and human olfactory mucosa indicate that basal cells exhibit distinct differences between the two species. These include basal cell morphology, marker expression and response to growth factors. In rodent HBCs and GBCs are morphologically distinct and HBCs express cytokeratin 14 whereas in human HBCs and GBCs are not morphologically distinguishable and express p75^NGFR^, an OEC marker [1,2,46,47]. It is claimed that human olfactory mucosa-derived spheres can be maintained in long-term in culture (up to 200 passages) whereas rodent olfactory mucosa-derived spheres have limited self-renewal potential and do not generate secondary spheres following passage [9,10,48]. EGF has predominantly survival effects on rodent HBCs and GBCs but no significant effect on human olfactory mucosa cell viability [9,49-52]. Thus, these differences caution against a too literal extrapolation from data obtained using rodent to the situation that will pertain in humans [53,54]. Nevertheless, we believe the broad principles established to be of translational value.

## Conclusion

By using media conditioned by olfactory-spheres made from the olfactory mucosa we have been able to test the ability of mucosa derived soluble factors to alter the properties of neural progenitor cells. Using neural progenitors from the olfactory bulb (a CNS structure containing progenitors of SVZ origin) we have shown that the olfactory mucosa does not produce factor that inhibit progenitor proliferation but neither does it contain factors that stimulate their proliferation compared to controls. However, we have shown that olfactory mucosa-derived factors stimulate neuronal differentiation of neural progenitors, favouring calretinin-containing rather than GABAergic phenotypes, and suppresses expression of GFAP by neural progenitor-derived astrocytes. Furthermore, the olfactory mucosa produces several factors with neuroprotective and neurotrophic properties. These data suggest that the difficulties encountered in expanding and propagating olfactory-spheres may be attributable to intrinsic properties of the olfactory epithelial stem/progenitor cells.

## Methods

### Animals

All the experiments were performed using neonatal CD1 mice (post-natal day 0 or P0 to post-natal day 3 or P3). All animal-related procedures were conducted in accordance with local ethical guidelines and approved animal care protocols.

### Olfactory mucosa conditioned medium preparation

Newborn mice were killed by decapitation and olfactory mucosae were immediately dissected into ice cold phosphate-buffered saline (PBS). Tissues were incubated for 20 min at 37°C into 0.25% Collagenase IA (Sigma) and 0.25% DNAse I (Roche) containing Dulbecco's Modified Eagle Medium (DMEM; Gibco) prior to mechanical dissociation to a cell suspension. Remaining cell aggregates were removed after filtration through a 40 μm cell strainer (BD Falcon) and cell numbers were estimated using the Trypan Blue exclusion method. Cell density was adjusted at ≈ 10^5 ^cells/ml. Cells were transferred into a poly 2-hydroxyethyl Methacrylate (PolyHEMA; Sigma) coated 25-cm^2 ^flask (Nunc) to avoid cell attachment in the "expansion medium" containing 1:3 mixture of DMEM/F12, 2% B27, 1% penicillin/streptomycin (P/S; all from Gibco), heparin (4 ng/ml, Sigma), supplemented with basic fibroblast growth factor (human recombinant bFGF; 20 ng/ml; R&D Systems), and epidermal growth factor (human recombinant EGF; 20 ng/ml; R&D Systems). Cultures were maintained for 14 days *in vitro *(div) at 37°C in a humid atmosphere with 95% oxygen and 5% CO_2_. Fresh medium was added to the cultures every third days. After 14 div, olfactory mucosa conditioned medium (OM-CM) was sterilized by filtration through a 0.22 μm membrane and stored at -20°C.

### Bulk olfactory bulb and subventricular zone neurosphere preparation

Newborn mice were killed by decapitation and the brains were immediately isolated into ice cold PBS. Brains were transferred into a new dissection dish containing clean ice cold PBS, meninges were removed prior to olfactory bulb and subventricular zone (SVZ) dissection. Bulbs and SVZ were mechanically dissociated to a cell suspension. Cell numbers were estimated using Trypan Blue exclusion method and cell density was adjusted at ≈ 10^5 ^cells/ml. Cells were transferred onto uncoated 25-cm^2 ^flask (Nunc) for 7 div in three different culture conditions. One culture condition consisted in 50% "expansion medium" (see composition above) – resulting in a final concentration of 10 ng/ml fresh bFGF and 10 ng/ml fresh EGF, 1% fresh B27 as for control 2 – and 50% OM-CM. Two control cultures were prepared named "control 1" in which the cells were plated in "expansion medium" (containing 20 ng/ml fresh bFGF, 20 ng/ml fresh EGF, 2% fresh B27), and a second control named "control 2" composed of 50% "expansion medium" and 50% DMEM/F12 – resulting in a final concentration of 10 ng/ml fresh bFGF, 10 ng/ml fresh EGF, and 1% fresh B27.

To induce differentiation, spheres from control cultures and OM-CM cultures were dissociated mechanically to a cell suspension prior to plating either onto laminin/collagen-coated 8 chamber slides (for immunofluorescence) or culture flask (for western blots or RT-PCR) at the density ≈ 10^5 ^cells/ml. Differentiation was induced by plating spheres expanded in OM-CM or control cultures in a medium supplemented with 1% fetal bovine serum (Sigma) instead of the growth factors and OM-CM. Cultures were maintained either for 3 div (to measure expression level of gene transcripts by RT-PCR) or for 7 div (for immunocytochemistry, RT-PCR, or western blots). After 7 div, cells were fixed for 15 min with 4% paraformaldehyde (PFA) containing PBS at room temperature (RT), rinsed three times in PBS and processed for immunocytochemistry.

### Immunocytochemistry

For double-immunolabelling differentiated neurosphere cultures were pre-incubated in 5% normal serum and 0.025% Triton-× in PBS ("incubation solution"), 1 h at RT prior to incubation overnight at RT with primary antibodies diluted in the incubation solution. The primary antibodies used were: mouse anti-Nestin (1:1000, Chemicon), mouse IgG_2b _anti-βIII-tubulin (1:250, Sigma), rabbit IgG anti-glial fibrillary acidic protein (GFAP; 1:500, DAKO), rabbit polyclonal anti-γ aminobutyric acide (GABA; 1:1000, Sigma), rabbit polyclonal anti-calretinin (1:500, Swant). After three rinses in PBS, cells were incubated with FITC- or Cy3-conjugated secondary antibodies (1:200, Jackson Immunoresearch) 2 h at RT. Cultures were mounted in 4',6-diamidino-2-phenylindole (DAPI) containing VECTASHIELD (Vector Laboratories).

### RT-PCR

RNA was isolated from olfactory-spheres expanded in EGF/bFGF for 14 div and from olfactory bulb neurospheres cultured either in OM-CM or in fresh medium (control 1 and 2). Total RNA from spheres was isolated using RNAqueous-Micro kit at the manufacturer recommendation (Ambion) and treated with DNase I prior to cDNA synthesis. RNA (100 ng) was reversed transcribed into cDNA using oligo-(dT)_12–18 _primers (Invitrogen) and Sensiscript Reverse Transcriptase according to the manufacturer's recommendations (Qiagen). Two microliters of cDNA were amplified in a thermal cycler using the following programs for each primer pairs: denaturation at 95°C for 1 min, annealing at a primer-specific temperature (see list of primers in Tables 1, 2, 3) for 1 min, followed by extension at 72°C for 1 min. PCR products were amplified using 35 cycles except for the cyclophilin (housekeeping gene) that were amplified using 25 cycles.

**Table 1:** List of primers for neural stem progenitor cell markers

**Genes**	**Forward Primer**	**Reverse Primer**	**Tm**	**References**
***Cyclophilin***	TGAGCACTGGGGAGAAAG	AGGGGAATGAGGAAAATATGG	51°C	modified from [55]
***Hes1***	CACGCTCGGGTCTGTGCTGAGAGC	ATGCCAGCTGATATAATGGAG	58°C	[56]
***Hes5***	GTGGAGATGCTCAGTCCCAAG	TGTAGTCCTGGTGCAGGCTC	55°C	[56]
***Nestin***	AGTGTGAAGGCAAAGATAGC	TCTGTCAGGATTGGGATGGG	54°C	[57]
***Notch1***	GGTGAACAATGTGGATGCTG	GTGGAGACAGAGTGGGTGT	52°C	[58]
***Sox2***	TTACCTCTTCCTCCCACTCC	TGATTGCCATGTTTATCTCG	56°C	Our laboratory

**Table 2:** List of primers encoding markers of bulbar interneuron proliferation and differentiation

**Genes**	**Forward Primer**	**Reverse Primer**	**Tm**	**References**
*Arx*	CAGCATTTGGCAGGCTCT	AGGATGTTGAGCTGCGTGAG	56°C	[59]
*Dlx5*	ATGACAGGAGTGTTTGACAG	CTAATAAAGCGTCCCGGAGG	56°C	[60]
*Mash1*	AGCAGCTGCTGGACGAGCA	CCTGCTTCCAAAGTCCATTC	56°C	[61]

**Table 3:** List of primers for neurotrophic/growth factors

**Genes**	**Forward Primer**	**Reverse Primer**	**Tm**	**References**
*aFGF*	ACCGAGAGGTTCAACCTGCC	GCCATAGTGAGTCCGAGGACC	58°C	Our laboratory
*Galectin-1*	TCAAACCTGGGGAATGTCTC	TCAGCCTGGTCAAAGGTGAT	52°C	[62]
*NGF*	ACCATCTCAGGCCTTTCCTT	TGTTGGGTGGCCTAGGTTAG	52°C	Our laboratory
*VEGFA*	GAGAGAGGCCGAAGTCCTTT	TTGGAACCGGCATCTTTATC	52°C	Our laboratory

### Western blot

Olfactory bulb neurospheres (controls 1 and 2, and OM-CM cultures) differentiated for 7 div were collected by centrifugation and incubated for 15 min in lysis buffer (Sigma) containing a cocktail of protease inhibitor (Sigma). Samples were then centrifuged at maximum speed for 10 min at 4°C and the supernatant was collected. Protein concentration was measured using a spectrophotometer. Five μg proteins were diluted in loading buffer containing DTT (20 mM) and denatured at 95°C for 10 min prior to loading on a 12% acrylamide gel (Bio-Rad). Electrophoresis was performed in running buffer (0.5 M Tris Base, 1.92 M glycine, 0.5% SDS) for 1 h at 100 V. Proteins were transferred on a nylon membrane in transfer buffer (25 mM Tris Base, 150 mM glycine, 0.05% SDS, 20% methanol) at 100 V for 3 h on ice. The membrane was then rinsed in TBS-Tween buffer (10 mM Tris Base, 150 mM NaCl, 0.1% Tween20) before blocking in TBS-Tween containing 5% powdered milk at RT for 1 h. Membrane was rinsed three times in TBS-Tween and then incubated at 4°C overnight with rabbit IgG anti-GFAP (1:1000, DAKO) and mouse IgG anti-GAPDH (1:5000; Cell signaling technology) in TBS-Tween-Milk. Membrane was rinsed in TBS-Tween and then incubated for 1 h at RT with anti-mouse immunoglobulins conjugated with HRP (Dako). After additional washes, protein bands on the membrane were detected using ECL revelation kit (Amersham Bioscience).

### Cell counting and statistical analysis

Photomicrographs (magnification times 20) were taken in approximately 7 random visual fields per well. For each marker, cell quantifications were performed in 3 to 4 wells per cell cultures from 3 to 4 separate cultures (generated from distinct dissections). Total numbers of DAPI-stained nuclei in addition to total numbers of cells expressing the phenotypic markers (GFAP, βIII-tubulin, GABA and Calretinin) or cells co-expressing two markers (βIII-tubulin/GABA and βIII-tubulin/Calretinin) were counted directly on photomicrographs. Differences between culture conditions were assessed using one-way analysis of variance (ANOVA) followed by Bonferroni's multiple comparison test. The standard error means were considered significantly different when the value of the variance P was <0.05. Significance was accepted at the 95% confidence level.

## Authors' contributions

PB performed the cell cultures, RT-PCR, cell quantifications, statistical analysis, and generated the figures. XH performed the immunocytochemistry on cell cultures and western blots. MAC and RJMF participated in study design, direction and supervision. RJMF and PB prepared the manuscript. All authors read and approved the final manuscript.
